# Nap improved game-related technical performance and physiological response during small-sided basketball game in professional players

**DOI:** 10.5114/biolsport.2023.116004

**Published:** 2022-06-01

**Authors:** Maher Souabni, Omar Hammouda, Mehdi J. Souabni, Mohamed Romdhani, Wajdi Souissi, Achraf Ammar, Tarak Driss

**Affiliations:** 1 Interdisciplinary Laboratory in Neurosciences, Physiology and Psychology: Physical Activity, Health and Learning (LINP2), UFR STAPS (Faculty of Sport Sciences), UPL, Paris Nanterre University, Nanterre, France; 2 Research Laboratory, Molecular Bases of Human Pathology, LR19ES13, Faculty of Medicine, University of Sfax, Sfax, Tunisia; 3 Physical Activity, Sport and Health, UR18JS01, National Observatory of Sports, Tunis, Tunisia; 4 Department of Training and Movement Science, Institute of Sport Science, Johannes Gutenberg-University Mainz, Mainz, Germany

**Keywords:** Sleep, Sports, Heart rate, Perceived exertion, Performance

## Abstract

The effect of 40-min nap (NAP) opportunity on psycho-physiological outcomes and technical performance during small-sided basketball game (SSG) was examined in ten professional basketball players. Nocturnal sleep and naps were monitored by actigraphic recording and sleep diaries. Nocturnal total sleep time (TST), time in bed (TIB), sleep efficiency (SE), sleep onset latency (SOL) and wake after sleep onset (WASO) were analyzed. Subjective sleep quality was assessed with visual analogue scale (VAS). Profile of mood state (POMS) and simple reaction time (SRT) were measured before and after Nap and no-nap (CON) conditions. During both test sessions, participants played 10-min SSG. Technical and tactical performances were assessed using Team Sport Assessment Procedure. Volume of play (VP), attack with ball (AB), efficiency index (EI) and performance score (PS) were determined. Heart rate (HR) was measured during SSG, and rating of perceive excretion (RPE) after. Lower HR (p ≤ 0.03, d ≥ 0.78) and RPE (p = 0.007, d = 1.11) were obtained in NAP compared to CON. There was no significant difference in TIB, TST, SE, WASO and VAS between CON and NAP conditions. AB, EI and PS were higher in NAP compared to CON (0.0002 ≤ p ≤ 0.001, 1.3 ≤ d ≤ 1.8). A significant reduction was observed in POMS’ fatigue (p = 0.005, d = -1.16, Δ = -53.6%), anxiety (p = 0.02, d = -0.9, Δ = -32.1%), anger (p = 0.01, d = -0.94, Δ = -30.3%), and an improvement in vigor (p = 0.01, d = 0.99, Δ = + 23.8%); which may reflect better readiness after nap and more concentration to start a game-situation. In summary, NAP reduced fatigue, anger, anxiety and enhanced vigor, allowing better technical and tactical performances during basketball SSG.

## INTRODUCTION

Basketball is considered as an intermittent high-intensity sport characterized by the repetition of short (≈ 2 seconds) high-intensity (i.e., average work-to-rest ratio of 1:3.6 during competition) movements (i.e., runs, shuffles, sideway runs and jumps) [1, 2]. Studies analysing the physiological determinants of success in basketball revealed the importance of both aerobic and anaerobic pathways [1–5]. Indeed, the limited recovery time between high intensity actions and the need to maintain a high quality of these actions to win matches highlights the need for a well-developed (i) anaerobic system to be able to perform short duration high-intensity movements and (ii) aerobic metabolism allowing rapid recovery between intense efforts [1, 4]. Moreover, basketball players should have a good aerobic capacity to cover a distance of approximately 7.5 km per match [1]. While, explosive actions, such as changes of direction, jumps, sprints and movements at maximum intensity, depend on the anaerobic routes [6, 7]. Further, it has been reported that aerobic capacity is strongly correlated with high-intensity running during matches, and has been identified as one of the determinants of repeated sprint ability [3]. Besides, due to its complexity and mixed physical-technical-tactical nature, the game of basketball requires great attention to determining the physical demands and the required technical skills [8]. Several studies investigated physical demands in basketball [6, 7, 9–12]. However, technical demands remain poorly investigated [13, 14]. In this context, small-sided games (SSG) are widely used by basketball coaches in an attempt to simultaneously develop technical and tactical skills under high physical loads [2, 5, 14–17]. Furthermore, International Basketball Federation (FIBA) has shown increasing interest in SSG with 3 × 3 (10 min gameplay) becoming an Olympic sport since Tokyo 2020 games.

Recently, a growing body of evidence suggests that napping enhances physical and cognitive performances. It has been reported that a diurnal nap opportunity improved endurance [18, 19] and short term physical performance (*i.e.,* jump [19], sprint [20], postural control [21] and strength [22, 23]). Interestingly, several studies showed improvement in repeated-sprint ability following various nap opportunities (*e.g.,* 20-min [24–26], 25-min [27, 28], 35-min [27], 40-min [23], 45-min [27]…). Due to the importance of repeated-sprint ability and the aerobic-anaerobic aspect of basketball during competition, it would be of interest to investigate the effect of napping on real-situation games.

The only study that investigated the effect of sleep extension on basketball athletes showed that the increase in sleep duration was associated with improvements in reaction time, sprint speed, shooting accuracy, free throw percentage, and three-point field goal percentage [29]. It is important to mention that naps were allowed in this study during control and sleep extension conditions; however, data about nap’s exact duration was not reported. Thus, it might be useful to measure nightly sleep episodes and daytime naps separately to get more insight about nap’s effect. In addition, the gamerelated technical aspect was not evaluated in this study [29]. To the best of authors’ knowledge, no study examined the effect of napping on technical performance during small-sided basketball games.

Therefore, the aim of the present study is to examine the effect of 40-min nap opportunity on cognitive outcomes and technical performance during SSG in professional basketball players. We hypothesized that diurnal napping would (i) improve technical game-related performance and (ii) enhance physiological response of athletes during SSG situations.

## MATERIALS AND METHODS

### Participants

Ten high-level professional basketball players (age, 27.6 ± 4.7 years; height, 194.2 ± 7.3 cm; mass, 89.6 ± 11 kg; body mass index, 23.4 ± 1.8 m · kg^-2^; body fat, 13.71 ± 2.5%, expertise, 18 ± 5 years, normative retiring and rising times: 22:57 ± 0:22 h: min and 07:00 ± 0:48 h: min, respectively) volunteered for this study. Verbal explanation of the experimental procedure was provided to each individual. Then they provided written informed consent before participating in the study. All of them were non-habitual nappers. They trained regularly 6 to 8 times per week (≈ 2 h/practice) since they signed a professional contract (*i.e.,* professional career, 9 ± 4 years). They were asked to sustain from consuming tobacco, alcohol or caffeine and were not allowed to nap during the experimental days. Based on the Horne and Ostberg self-assessment Morningness–Eveningness Questionnaire (MEQ) [30], participants who presented an extreme morning or extreme evening type were eliminated (MEQ mean score = 56 ± 3.1). The protocol of the present study was approved by the local Institutional Review Board (CPP SUD N° 0339/2021) and complied with Helsinki’s declaration for human experimentation.

### Procedure

**FIG. 1:**
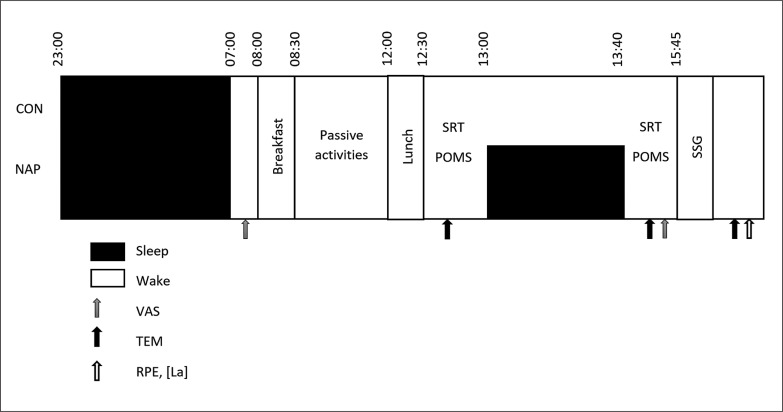
Experimental protocol. ESS indicates Epworth Sleepiness Scale; POMS, Profile of Mood State; RPE, rating perceived of exertion; SRT, simple reaction time; TEM, temperature; VAS, visual analog scale; [La], blood lactate concentration.

A randomized crossover design was used. The participants were asked to visit the physiology laboratory on three separate occasions. During the first visit, anthropometric data were collected and participants were familiarized with the experimenters, laboratory, used material/ devices, the sleeping room, tests, questionnaires and the experimental procedures. The experimental design is described in Figure 1.

Two weeks after the familiarization session, 2 test sessions were undertaken, 72 h apart, after normal sleep night. In both test sessions, participants arrived at the laboratory at 7:30. They subjectively rated their last night sleep on a visual analog scale and ate a qualitatively and quantitatively standardized breakfast at 08:00 h. Then participants stayed awake doing passive activities (*e.g.,* surfing on the internet, watching television, playing video games, reading) until noon; when they ate an isocaloric lunch. Afterwards, they were assigned to experience both nap (NAP) and no-nap (CON) conditions (*i.e.,* 13:00 h – 13:40 h) in a randomized order. Before and after each condition, simple reaction time (SRT) and intra-aural temperature (TEM) were measured and the profile of mood state questionnaire (POMS) was administrated.

In the NAP condition, participants entered the comfortably warm, fully dark, and quiet sleeping room at 12:45 h. Nap opportunity started at 13:00 h, after 10 min of acclimatization in bed, and lasted 40 min. The nocturnal sleep and naps were monitored by actigraphic recording and sleep diaries. In the CON condition, participants spent the same time (*i.e.,* 13:00 h – 13:40 h) seated on a comfortable chair watching television.

In both conditions, participants completed a 15 min standardized warm-up at 15:30 h. The warm-up consisted of a 5 min jog at a self-selected comfortable pace, followed by a 10 min of dynamic stretching (*e.g.,* lunges, butt kicks, hip flexion/extension, hip abduction/adduction), and five progressive sprints. Then, participants played a 10 min SSG. Heart rate (HR) was assessed during SSG, and blood lactate concentration [La], rating of perceived exertion (RPE) and TEM were assessed after the game for both NAP and CON conditions.

### Measured Variables

#### Eliteness of athletes

Eliteness’ mean score was 9.16 ± 1.2. Based on Swan et al.[31]’ categories, athletes belong to the second-highest qualification, i.e., successful elite.

### Actigraphy and sleep diaries

For objective sleep measurements, participants wore GT3X activity monitors on their non-dominant arm (Actigraph, Pensacola, FL, USA), the night before each experimental day and during daytime napping opportunities. This non-invasive and cost-effective device was evaluated as a valid tool to assess sleep and wake behaviors compared to the gold standard polysomnography [32]. The GT3X data was downloaded and analyzed using Actilife 6 (version 6.13.7) software. A high-level sensitivity was used for the estimation of sleep-wake patterns in 60-s epochs. Actigraphic records were edited with information listed in the subjective sleep diaries. The following sleep parameters were derived and analyzed: total sleep time (TST), time in bed (TIB), sleep efficiency (SE), sleep onset latency (SOL) and wake after sleep onset (WASO).

### Subjective sleep quality

The subjective sleep quality was evaluated using the visual analogue scale (VAS) [33]. An important related advantage of VAS is that, unlike whole numbers and words, they provide an unlimited number of possible responses along a single continuum [34]. The sleep quality evaluation – VAS scale is a 10-cm scale. The scale shows “Very bad sleep quality” on the left side, and “Very good sleep quality” on the right side. The sleep value of the VAS is determined by measuring the distance between the marked point and the far-left end of the scale. VAS was administrated after both, nocturnal sleep and nap opportunity.

### Team sport assessment procedure (TSAP)

TSAP has been used widely by students, teachers, coaches and researchers [35, 36]. In basketball, TSAP’s validity was ascertained through inter-observer reliability and test-retest method [37]. Further, TSAP was used to analyze the effect of different SSGs on technical and tactical performance among youth basketball players of different levels, i.e., U14 and U16 [13].

TSAP focus on the offensive on-ball aspects of the game, assessing how a player gains the balls possessions, recording the number of Conquered Balls (CB) and Received Balls (RB); and how a player disposes of the ball, recording the number of Lost Balls (LB), Neutral Balls (NB), Influent Passes (P) and Success Shots (SS) (Table 1).

**TABLE 1:** Team sport assessment procedure components of Game Play

Components	Definitions
**Gaining possession of the ball**	
Conquering the ball (CB)	Interception. Stealing the ball from the opponent, or recapturing the ball after an unsuccessful shot on goal or near loss to the other team.
Receiving the ball (RB)	Receiving the ball from a teammate and not immediately losing control of it.

**Disposing of the Ball**	
Playing a neutral ball (NB)	Passing the ball to a teammate, or any pass that does not put the other team in jeopardy.
Losing the ball (LB)	Losing the ball to the other team without having scored a goal.
Influent passes (P)	Passing the ball to a partner, thus pressuring the other team, which most often leads to a shot on goal.
Executing a successful shot (SS)	Scoring or maintaining possession of the ball following the execution of a shot.

Note: Adapted from Gréhaigne et al. [36].

Observational analysis was conducted after video collection by two authors (MS and MJS), separately, using a sample of a variable recording sheet [36]. The authors that performed TSAP analysis had between 15 and 20 years of basketball’s experience. All the variables were computed and used to calculate the indexes of “Volume of Play”, “Attack with ball”, “Efficiency index” and “Performance Score”, using different formulas (Table 2). Cohen’s k coefficient value (k = 0.86) reveals an almost perfect inter-observer agreement among all the observations [36].

**TABLE 2:** Formulas for calculating team sport assessment procedure variables outcomes.

Outcome variables	Calculation
Volume of play (VP)	CB + RB
Attack with ball (AB)	P + SS
Efficiency index (EI)	AB / (10 + LB)
Performance score (PS)	(VP /2) + (EI × 10)

Abbreviations: CB = conquering the ball; LB = losing the ball; P = Influent Passes; RB = receiving the ball; SS = executing a successful shot; VP = volume of play.

### Profile of mood state (POMS)

POMS standard validated psychological test [38] was administered pre and post nap/rest. It consists of 65-items classified into seven different subscales; anxiety-tension, anger-hostility, confusion-perplexity, depression-discouragement, fatigue-inertia, vigor-activity and interpersonal relationship.

### Intra-aural temperature (TEM)

TEM was measured using ThermoScan^®^ 7 (Braun, IRT6520WE, Germany) equipped with Age Precision^®^ technology which provides more precise temperature measurements depending on age. TEM was measured pre and post nap/rest, and at the end of SSG.

### Simple reaction time (SRT)

SRT was assessed using software Reaction, INRP free software (4.05 version). Participants were asked to respond as quickly as possible to a visual stimulus (Blue Square) presented systematically at the center of the computer screen. SRT was measured before and after nap/rest.

### Heart rate (HR)

The physiological demand was assessed using HR monitor chest belts (Team System 2, Polar, Kempele, Finland). HR beats were exported and analyzed using Excel software (Microsoft Corporation, Redmond, WA, USA). SSG’ HR data were expressed as mean (HR_mean_) and peak (HR_peak_) HR as a percentage of each subject’s individual HR_max_ (%HR_max_).

### Rating of perceived exertion (RPE)

RPE scores were collected at the end of SSG using the Borg 15-grade scale [39], which is considered a valid method to evaluate players’ training load in team sports and has been widely used during basketball ball-drills [2, 14]. This psycho-physiological scale given score represents the exertion that the athlete experiences during the exercise [40].

### Blood lactate concentration [La]

[La] was measured at the end of SSG using the Lactate Pro 2 portable blood lactate meter (Arkray, Kioto, Japan) on micro blood samples drawn from the tip of the index finger according to the manufacturer’s instructions.

### Statistical analysis

The following analyses were performed using Excel (Microsoft Office, v.2016) and SPSS Statistics (IBM, v.23) softwares. All data were expressed as means ± standard deviations (SD).

The Shapiro-Wilk W-test revealed that TEM, POM’S variables, VP, AB, PS, EI, HR_mean_, HR_peak_, RPE and [La] were normally distributed. TEM and POMS were analyzed using a two-way repeated-measures ANOVA [2 conditions (CON and NAP) × 2 times (pre and post rest/nap)] for POM’S, [2 conditions (CON and NAP) × 3 times (pre and post rest/nap and after SSG)] for TEM. All ANOVA effect sizes were calculated as partial eta squared (*np*^2^). When significant main or interaction effects were observed, pairwise comparisons were performed using Bonferroni post-hoc test.

Paired samples t-test was used to determine differences of VP, AB, PS, HR_mean_, HR_peak_, RPE and [La] between CON and NAP conditions. Effect sizes were calculated using Cohen (*d*) test [41]. Wilcoxon test was used to compare differences between CON and NAP conditions for SOL, SE, TIB, TST, WASO and VAS. Statistical significance level was set at p < 0.05.

## RESULTS

### Objective and subjective sleep Parameters

There was no significant difference during the night before experimental days in TIB (p = 0.3), TST (p = 0.5), SE (p = 0.5), SOL (p = 0.4), WASO (p = 0.3) and VAS (p = 0.5) between CON and NAP.

### TSAP

Although no significant difference was reported in VP, statistical analysis revealed a significant improvement for AB, EI and PS in NAP compared to CON condition (Table 3).

**TABLE 3:** Mean values (± SD) for observational variables of technical and tactical performance collected by the Team Sport Assessment Procedure.

		Mean ± SD	P	*d*	Δ (%)
VP	CON	21.70 ± 2.79	NS	–	–
	NAP	23.40 ± 4.01			

AB	CON	5.80 ± 2.20	0.001	1.57	+ 45.3
	NAP	10.60 ± 3.34			

EI	CON	0.33 ± 0.12	0.0002	1.80	+ 54.8
	NAP	0.73 ± 0.25			

PS	CON	14.16 ± 2.15	0.003	1.30	+ 25.3
	NAP	18.96 ± 3.86			

Abbreviations: AB = attack with ball; CON = control condition; EI = Efficiency index; NAP = nap condition; PS = performance score; VP = volume of play.

### POMS

Two-way repeated measures ANOVA revealed a significant interaction for anxiety (F_(1,9)_ = 17.8, p = 0.002, *ɳp*^2^ = 0.66), anger (F_(1,9)_ = 11.5, p = 0.008, *ɳp*^2^ = 0.56), depression (F_(1,9)_ = 9.9, p = 0.01, *ɳp*^2^ = 0.52), fatigue (F_(1,9)_ = 16.5, p = 0.003, *ɳp*^2^ = 0.64), and vigor (F_(1,9)_ = 6.8, p = 0.02, *ɳp*^2^ = 0.42). Scores are presented in Table 4.

**TABLE 4:** Mean (± SD) values for POMS’ scores (anger, anxiety, confusion, depression, fatigue, and vigor) before and after nap and control conditions.

	CON		NAP	
	Pre	Post	Pre	Post
Anger, au	14.0 ± 7.27	15.1 ± 7.03	17.8 ± 6.32	12.4 ± 4.33 **
Anxiety, au	9.0 ± 4.97	12.5 ± 3.98 **	10.6 ± 5.32	7.2 ± 4.71 **, ##
Confusion, au	9.4 ± 5.13	9.5 ± 4.74	10.3 ± 4.79	8.9 ± 4.31
Depression, au	7.0 ± 5.23	9.0 ± 6.58 *	8.0 ± 5.96	4.9 ± 3.11
Fatigue, au	8.5 ± 5.46	11.5 ± 5.5 **	11.0 ± 5.16	5.1 ± 2.81 **, ##
Vigor, au	17.4 ± 5.5	15.2 ± 6.37	16.1 ± 4.53	20.6 ± 4.30 **, #

Significant difference compared to pre nap/rest:

* P < 0.05,

** P < 0.01. Significant difference compared to control (CON) condition,

# P < 0.05,

## P < 0.01.

Scores were significantly lower after nap compared to values before nap for anxiety (p = 0.02, *d* = -0.9, Δ = -32.1%), anger (p = 0.01, *d* = -0.94, Δ = -30.3%) and fatigue (p = 0.005, *d* = -1.16, Δ = -53.6%). In addition, POMS’ fatigue (p = 0.002, *d* = -1.32, Δ = -55.7%) and anxiety (p = 0.003, *d* = -1.26, Δ = -42.4%) values were significantly lower after NAP compared to values after CON condition. In CON condition, Bonferroni post-hoc test revealed an increase in fatigue (p = 0.01, *d* = 0.9, Δ = + 29.2%), anxiety (p = 0.005, *d* = 1.2, Δ = + 30.8%) and depression (p = 0.04, *d* = 0.8, Δ = + 22.2%) scores after CON compared to values before CON. Furthermore, vigor’ scores improved after nap compared to both, pre nap (p = 0.01, *d* = 0.99, Δ = + 23.8%) and after CON (p = 0.03, *d* = 0.8, Δ = + 28.6%) values.

### TEM

Repeated measure ANOVA revealed a significant effect of time (F_(2,18)_ = 69.5, p < 0.0005, *ɳp*^2^ = 0.88) and a significant interaction (F_(2,18)_ = 12.3, p < 0.0005, *ɳp*^2^ = 0.59).

**FIG. 2:**
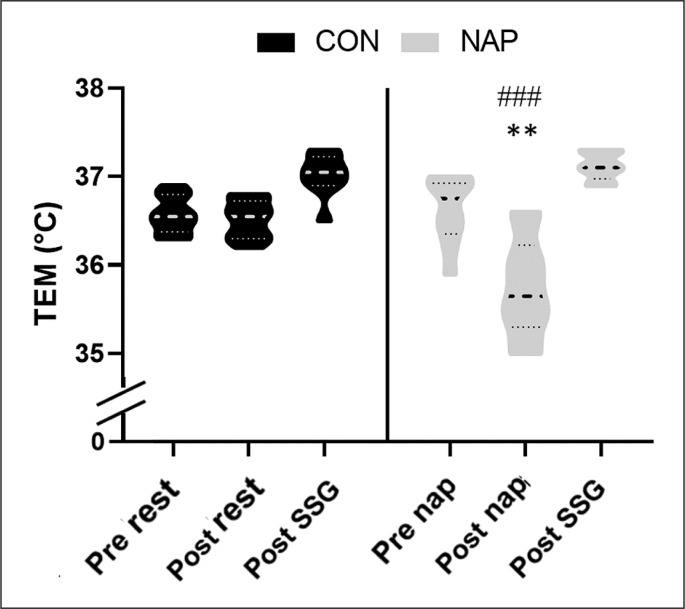
Violin plot for intra-aural temperature (TEM) before and after nap/rest and after SSG in CON and NAP conditions. Significance was assessed with Two-way repeated-measures analysis of variance and Bonferroni post hoc tests. Horizontal and vertical bars represent the group means and SDs, respectively. SSG indicates small sided games. Significant difference compared to CON condition: **P < 0.01. Significant difference compared to pre nap/rest: ###P < 0.001.

Bonferroni post-hoc test revealed that TEM was significantly lower in NAP after nap/rest compared to pre nap (p = 0.001, *d* = 1.61, Δ = 2.4%) and compared to CON condition (p = 0.005, *d* = 1.16, Δ = 2.1%) (Figure 2).

### SRT

There was no significant effect of conditions (F_(1,9)_ = 0.5, p > 0.05, *ɳp*^2^ = 0.006) or time (F_(1,9)_ = 0.03, p > 0.05, *ɳp*^2^ = 0.05) on SRT.

### HR

**FIG. 3:**
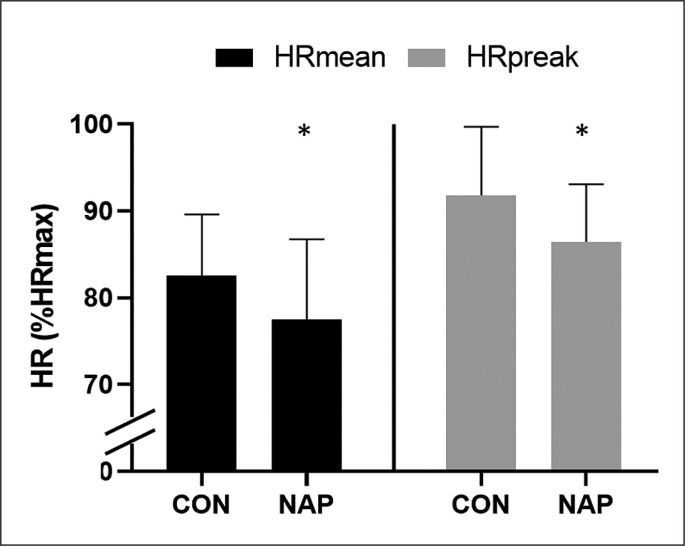
Mean values (± SD) for HR_mean_ and HR_peak_ during SSG in CON and NAP conditions. Significant difference compared to CON condition: *P < 0.05.

The pairwise comparison revealed a significant effect of nap on HR. Results showed lower values in NAP compared to CON condition for HR_mean_ (p = 0.03, *d* = 0.78, Δ = 6.2%) and HR_peak_ (p = 0.02, *d* = 0.83, Δ = 5.8%) (Figure 3).

### RPE

**FIG. 4:**
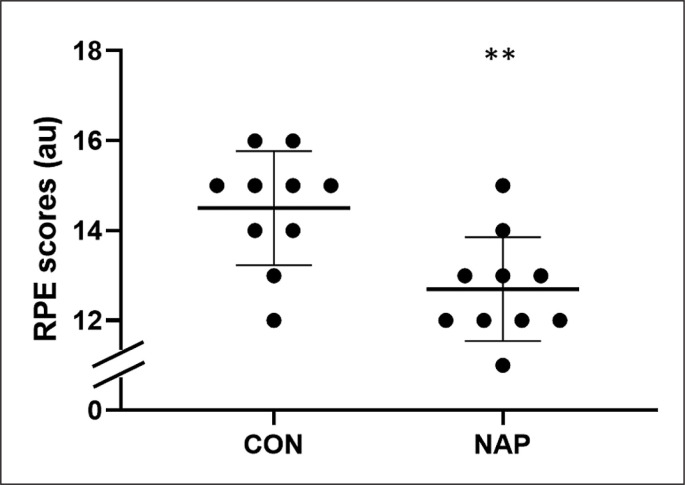
Individual values for RPE scores in CON and NAP conditions after SGG. Significant difference compared to CON condition: **P < 0.01.

Statistical analysis revealed a significant effect of nap on RPE scores. Results showed lower values in NAP compared to CON condition (p = 0.007, *d* = 1.11, Δ = 12.4%) (Figure 4).

### [La]

The paired Student t-test did not reveal a significant effect of nap on [La].

## DISCUSSION

Although napping is commonly used as a strategy to improve human’s physical and cognitive performances during separated exercise, to our knowledge, the present study is the first to evaluate the effectiveness of napping on (i) technical and tactical performance and (ii) physiological responses during real-situation game. Accordingly, the main findings of the current study were that NAP improved observational variables (AB, EI and PS) and physiological responses, decreased RPE, anger, fatigue, anxiety and intraoral temperature, and increased vigor. Importantly, in addition to the mean score of eliteness (i.e., 9.16 ± 1.20), body fat percentage (i.e., 13.7%) gives an additional argument regarding the high level of the athletes included in the present study. Indeed the average percentage of professional basketball players’ body fat for men is 13.5 ± 2.9% [42].

There was no significant difference during the night before experimental days in sleep parameters (i.e., TIB, TST, SE, SOL and WASO) and subjective sleep quality – VAS the night preceding testing between CON and NAP conditions. About 7 h of TST and 85% of SE was obtained in each condition, which is the lowest recommended sleep duration for athletes (7–9 h; [43]) and the lowest SE recommended for healthy adults to promote health (85%; [44]. Indeed, athletes require more sleep than the general population due to the imposed training load [45]. Evidence suggest that 8–9 h of TST are needed for athletes for optimal waking functioning [29, 46].

Interestingly, a major result in this study was that NAPO improved AB, EI and PS, while no significant difference was reported for VP between both conditions. It is important to mention that VP is the sum of conquered balls and received balls. As mentioned above TSAP focus on the offensive on-ball aspects of the game, assessing (i) how a player gains the balls possessions, recording the number of conquered balls and received balls and (ii) how a player disposes of the ball, recording the number of lost balls, neutral balls, influent passes and success shots. Therefore, the results of the present study showed that NAPO had a positive impact on the part of how the player disposes of the ball by improving AB, EI and PS. In this vein, according to Lastella et al. [47], the ability to make fast and accurate decisions in team sports is just as important as executing skills efficiently during competitions. Accordingly, our results are probably due to better decision making [48] and mastery of technical skills [49]; which implies better control of the ball, less turnovers and less mistakes on offense.

Otherwise, our results showed that RPE mean values during SSG were 12.4% lower after NAP compared to CON condition. These results are essentially the same as previous studies that reported a decrease in RPE mean values by 12.4% [23] and 16.7% [50] following 40-min NAPO and 19.6% following 45-min NAPO [27]. It is important to mention that shorter NAPO (i.e., 20 ≤ NAPO ≤ 35 min) had minor (i.e., 1 ≤ Δ RPE ≤ 6%) [27] or even no effect on RPE scores [24, 28]. Thus, it could be posited that the participants performed better in NAP because they perceived the exercise to be less strenuous compared to CON condition. Moreover, in the present study RPE results were confirmed by a decrease in HR mean values, i.e., HR_mean_ and HR_peak_ (by 6.2% and 5.8%, respectively). To our knowledge, this is the first study to investigate the effect of daytime napping on HR response during physical effort. Further, we did not report any significant effect of nap on [La] measured after SSG. Prior investigations revealed higher [La] following 20 min NAPO after PSD [24] and NSN [51]; whereas, in the line with our results, no significant effect was reported following 90 min NAPO for both studies [24, 51]. It is possible that longer NAPO (i.e., NAPO ≥ 40 min) resulted in a greater aerobic contribution in energy synthesis compared to short NAPO [51]. In view of these results, it could be argued that improvement of technical and tactical performance during SSG is likely due to the better physiological response. This may increase the active recovery, and thus prepare the players for high intensities and to restart in the following offensive process. In this context, it was suggested that non-rapid eye movement (NREM) sleep is the time in which body actively repairs and restores itself [52]. This could explain the positive impact of napping on physiological response. Additionally, slow wave sleep – also known as deep NREM sleep – is thought to play an important role in cerebral restoration and recovery [53], which might explain the improvement of decision making.

Furthermore, mood states estimated by the POM’S (i.e., fatigue and anxiety) were significantly lower after NAP compared to CON. Additionally, nap improved vigor scores. In this context, it has been reported that mood state and physical performance have a direct relationship with sleep quality and quantity [23, 24, 51]. In agreement with presents findings, Boukhris et al. [23] revealed that 40 min NAPO decreased fatigue (Δ = 31.7%) and anxiety (Δ = 27.5%), and increased vigor (Δ = 16.6%) of fourteen amateur team sport players. Thus, the improvement of technical and tactical performance during the SSG after nap could also be explained by a reduction in fatigue and an increase in vigor. Importantly, for the same NAPO duration (i.e., 40 min), we reported higher percentages of improvement 55.7%, 42.4% and 28.6% (for fatigue, anxiety and vigor, respectively). This could be related to the fact that participants in our study are professional basketball players and probably carrying a greater sleep dept compared to participants in Boukhris et al. [23] study. In fact, athletes are known to experience chronically shortened sleep duration [54]. Elite athletes’ sleep may be more disturbed than normal due to reasons such as jetlag, altitude, early morning training, increases in training load and traveling to sport meetings which might involve getting up early in the morning or retiring late at night [55]. In addition, a recent systematic review focused on the effect of sleep extension in athletes and concluded that the most important factor is athlete’s normal sleep pattern and whether he/she is getting adequate sleep [56].

Besides, despite being in the ascending phase of temperature’s circadian rhythm, current data showed a marked drop in TEM following NAPO. These results are in the line with prior investigations showing that napping decreased body temperature [20, 24, 51]. In this context, it has been reported that starting exercise with a lower core body temperature increases the duration of the exercise and delays fatigue onset [57]. In fact, hyperthermia causes a decrease in cerebral perfusion, which alters the evacuation of heat from the brain and reduces cerebral oxygenation during exercises [58]. However, in the present study we didn’t have a significant effect of nap on TEM after SSG. The lack of a significant difference between the conditions could be related to the short duration of exercise (i.e., 10 min). Therefore, napping could be suggested as precooling technique to enhance endurance performance during long duration exercises. Nevertheless, further researches are required to investigate thermoregulatory response including long duration exercises.

### Limitations

First, a small sample size was included in the present study. Second, napping is often seen as a response to insufficient nocturnal sleep, the current study shows a positive effect of napping in professional basketball players with normal sleep. However, it is well known that sleep disturbances are frequent amongst athletes, which implies poorer sleep duration and lower sleep quality [55]. Thus, these results cannot be systematically generalized to all populations, and larger studies are still needed to confirm the veracity of the present findings. Third, the selected participants were non-habitual nappers. It was reported that non-habitual nappers displayed heavier sleep inertia at the awakening compared to habitual nappers [59]. Thus, results could be different if habitual nappers were included instead of non-habitual nappers especially for SRT in which no significant result was reported. Finally, future researches should consider the use of objective sleep measures (e.g., polysomnography) during nap opportunities in order to get more insight of sleep stages and nap duration.

## CONCLUSIONS

The results of the present study showed that 40 min nap opportunity has a beneficial effect on technical and tactical performance, physiological responses and perceived exertion during a real game situation among elite athletes. In addition, napping may be an effective strategy for reducing fatigue, anxiety, anger and TEM, and enhancing vigor following a reference night. From a practical point of view, these findings can be placed in a professional context by implementing napping in athletes’ schedule. Accordingly, basketball players are advised to plan a nap before practice and/or competition in order to improve their technical and tactical performance. Noting that, these results may suit the players’ goals, and without violating doping regulations.
